# Supplementary figures and images for: Social capital as a network measure provides new insights on economic growth
Source: PLoS One. 2022 Aug 26;17(8):e0273066. doi: 10.1371/journal.pone.0273066 (PMC9417001; doi:10.1371/journal.pone.0273066)

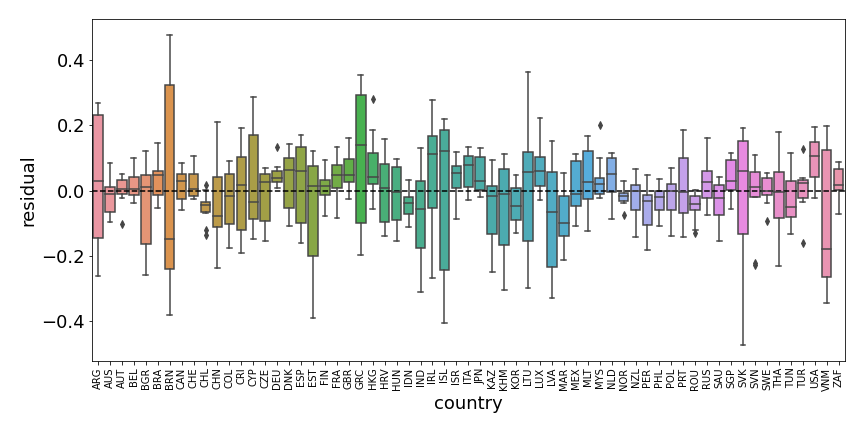

Supplement: S1 Fig — We observe homoskedasticity of the residuals across different countries. (TIF) [file pone.0273066.s001.tif]

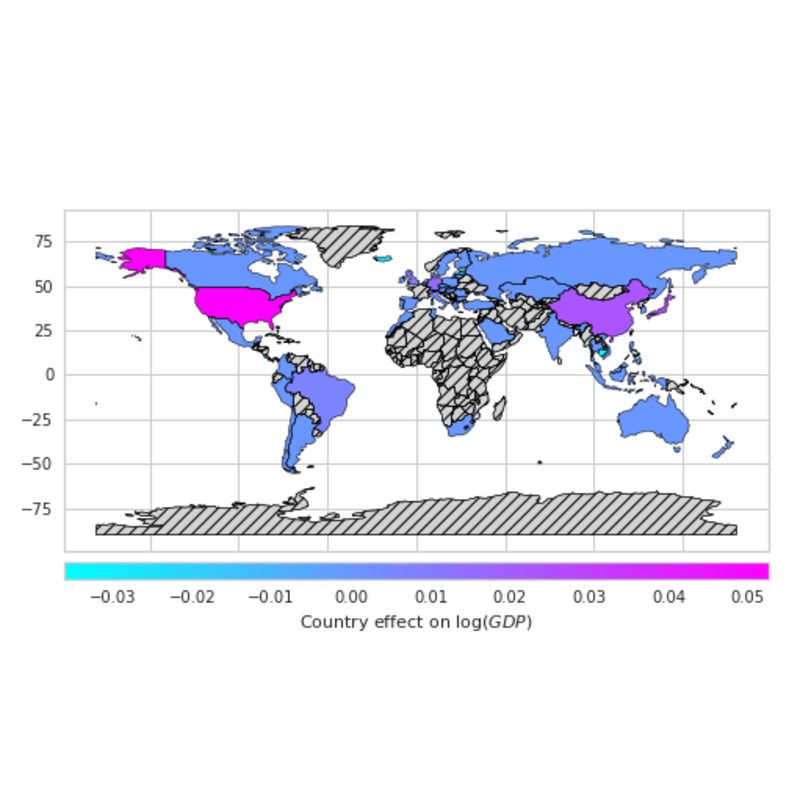

Supplement: S2 Fig — Elasic-Net estimates World Map containing the country level fixed effects coefficients estimated with the Elastic-Net regressor. We observe excess economic output for the US, China and Japan. (TIF) [file pone.0273066.s002.tif]

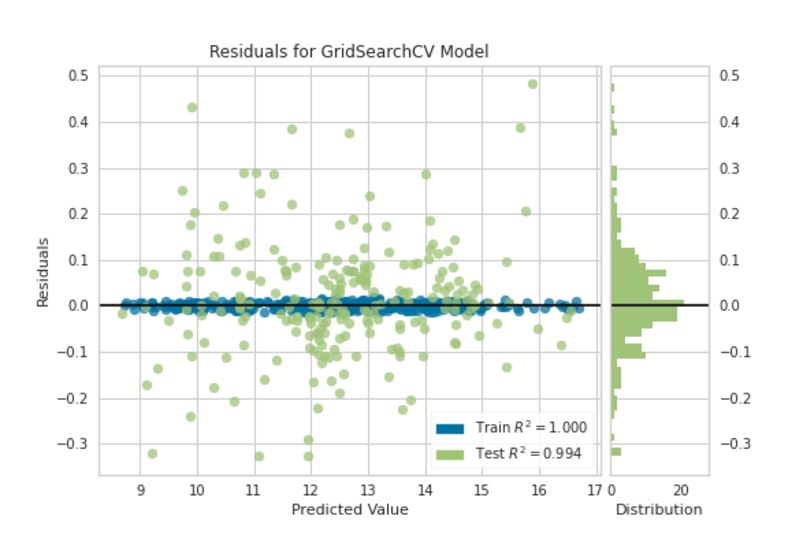

Supplement: S3 Fig — Gradient Boosting Regressor model residuals for both the training and test sets. We observe very high model performances in both, as well as homoskedasticity of the residuals. (TIF) [file pone.0273066.s003.tif]

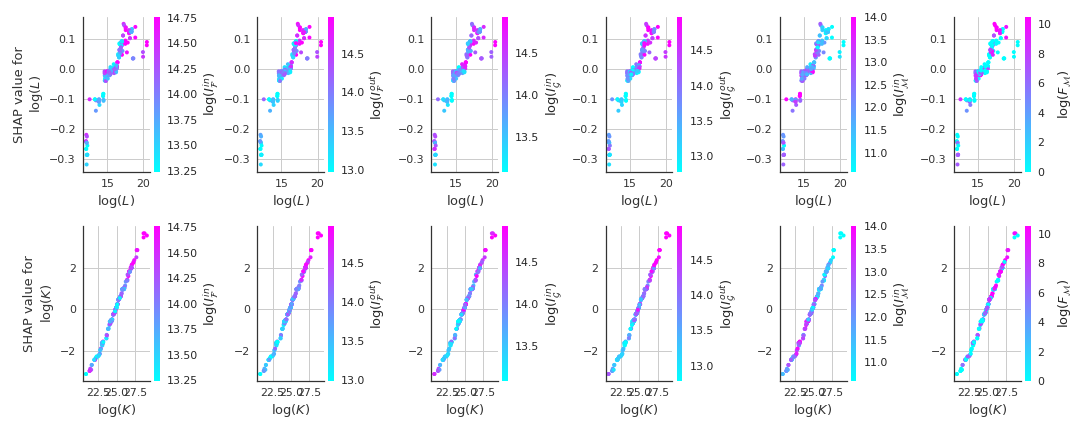

Supplement: S4 Fig — SHAP interaction effects for the different features in the Gradient Boosting Regressor. We detect strong interaction patterns between the social capital, and capital and labor respectively. This result provides evidence for the existence of interaction effects between the model variables. (TIF) [file pone.0273066.s004.tif]
