# Supplementary material for: Social capital as a network measure provides new insights on economic growth
Source: PLoS One. 2022 Aug 26;17(8):e0273066. doi: 10.1371/journal.pone.0273066 (PMC9417001; doi:10.1371/journal.pone.0273066)
Supplement: Attachment — Submitted filename: Review Report_Social capital as a network measure provides new insights on economic growth.pdf [file pone.0273066.s005.pdf]

Review report for the paper “**Social capital as a network measure provides new insights on economic growth**”

<u>Comments</u>

Using a complex systems approach, from Multi-Regional Input-Output and International Migration datasets, they build a network that allows them to examine the role of social capital as a driver of total factor productivity. The document is clear, well written, and the topic is relevant and current. At the same time, the approach and techniques used for the analysis are pertinent and relatively new to the economics discipline.

I recommend accepting the publication

Additional comment (to the editor): I don't know if it was by mistake or if it is a common practice of your prestigious journal, it caught my attention that the manuscript that I reviewed contained all the data of the authors. Although I have no link of any kind with them and therefore my evaluation was not biased in any way, I do not think it is the most convenient. Although I have no link of any kind with them and therefore my evaluation was not biased in any way, I do not think it is the most convenient.
